# Supplementary material for: Effects of Magnesium Oxide and Magnesium Hydroxide Microparticle Foliar Treatment on Tomato PR Gene Expression and Leaf Microbiome
Source: Microorganisms. 2021 Jun 4;9(6):1217. doi: 10.3390/microorganisms9061217 (PMC8228823; doi:10.3390/microorganisms9061217)
Supplement: Supplementary file 1 [file microorganisms-09-01217-s001.zip › microorganisms-1208996-supplementary.pdf]

Supplementary Information Document

Effects of magnesium oxide and magnesium hydroxide microparticle foliar treatment on tomato PR gene expression and leaf microbiome.

Aggeliki Andreadelli [1], Spyros Petrakis [1], Antiopi Tsoureki [1], George Tsiolas [1], Sofia Michailidou [1], Penelope Baltzopoulou [2] , Robert van Merkestein[3], Philip Hodgson[3], Mark Sceats[3], George Karagiannakis [2] and Antonios M. Makris [1,*]

May 2021

*corresponding author: makris@certh.gr; Tel.: +30-2311-257-541

The file includes:
Table S1: Primer sequences for NGS libraries
Table S2: Primer sequences for qPCR 5'-3'
Figure S1: Energy-dispersive X-ray spectroscopy of sprayed tomato leaves

Primer sequences

Table S1. Primer sequences for NGS libraries

16S_F	5′-TCGTCGGCAGCGTCAGATGTGTATAAGAGACAGCCTACGGGNGGCWGCAG-3′
16S_R	5′-GTCTCGTGGGCTCGGAGATGTGTATAAGAGACAGGACTACHVGGGTATCTAATCC-3′
18S_F	5′-TCGTCGGCAGCGTCAGATGTGTATAAGAGACAGCGATAACGAACGAGACCT-3′
18S_R	5′-GTCTCGTGGGCTCGGAGATGTGTATAAGAGACAGANCCATTCAATCGGTANT-3′

Table S2. Primer sequences for RT-qPCR 5'-3'

		RT-qPCR product	Accession	Source reference
	beta-1,3-glucanase (PR2)			
Forward	5'-GGACACCCTTCCGCTACTCTT-3'	81bp	M80604	[1]
Reverse	5'-TGTTCCTGCCCCTCCTTTC-3'			
	glucan endo-1,3-beta-glucosidase B (PR2B)			
Forward	5'-CCCATTTCAAGTTCCTGCTT-3'	112bp	M80608.1	[2]
Reverse	5'-AGAATTGCCAATCAACGTCA-3'			
	CHI9 chitinase (PR3)			
Forward	5'-AACTATGGGCCATGTGGAAGA-3'	128bp	Z15140	[1]
Reverse	5′-GGCTTTGGGGATTGAGGAG3'			
	CHI3 chitinase			

Forward<br>Reverse	5'-GGTTCTGGATGACAGAACAGGA-3'<br>5'-GTACCCTGGAACTCTATTAGCTGC-3'	107bp	Z15141	[3]
	Phenylalanine ammonia-lyase (PAL-4)			
Forward<br>Reverse	5′- ACGGGTTGCCATCTAATCTG-3′<br>5′-AGCTCTTTTCCTGGCTGAAA -3′	197bp	TIGRTC153699	[4]
	Elongation factor 1 alpha (EF1a)			
Forward<br>Reverse	5′-CTCCAAGGCTAGGTATGATGA-3'<br>5'-ACAGTTCCAATACCACCAATCT-3'	263bp	X14449.1	[5]

Energy-dispersive X-ray spectroscopy of sprayed tomato leaves

The Scanning Electron Microscopy (SEM) analysis of sprayed leaves, shown in the images of Figure 7 of the manuscript, was complimented by Energy dispersive X-Ray Spectroscopy (EDS) to verify the presence of magnesium (Mg) on the leaves surface. Representative images of EDS spectra from both Day 5 and Day 12 are provided in Figure S1 below. The main observations relate to the clear presence of Mg on the leaves sprayed with the 3 PMP slurries. The control sample (sprayed with distilled water) shows only a small background peak, while the intensity of the Mg peak is clearly higher in Day 5 as compared to Day 12, thereby confirming that the material was gradually removed from the leaves surface with time.

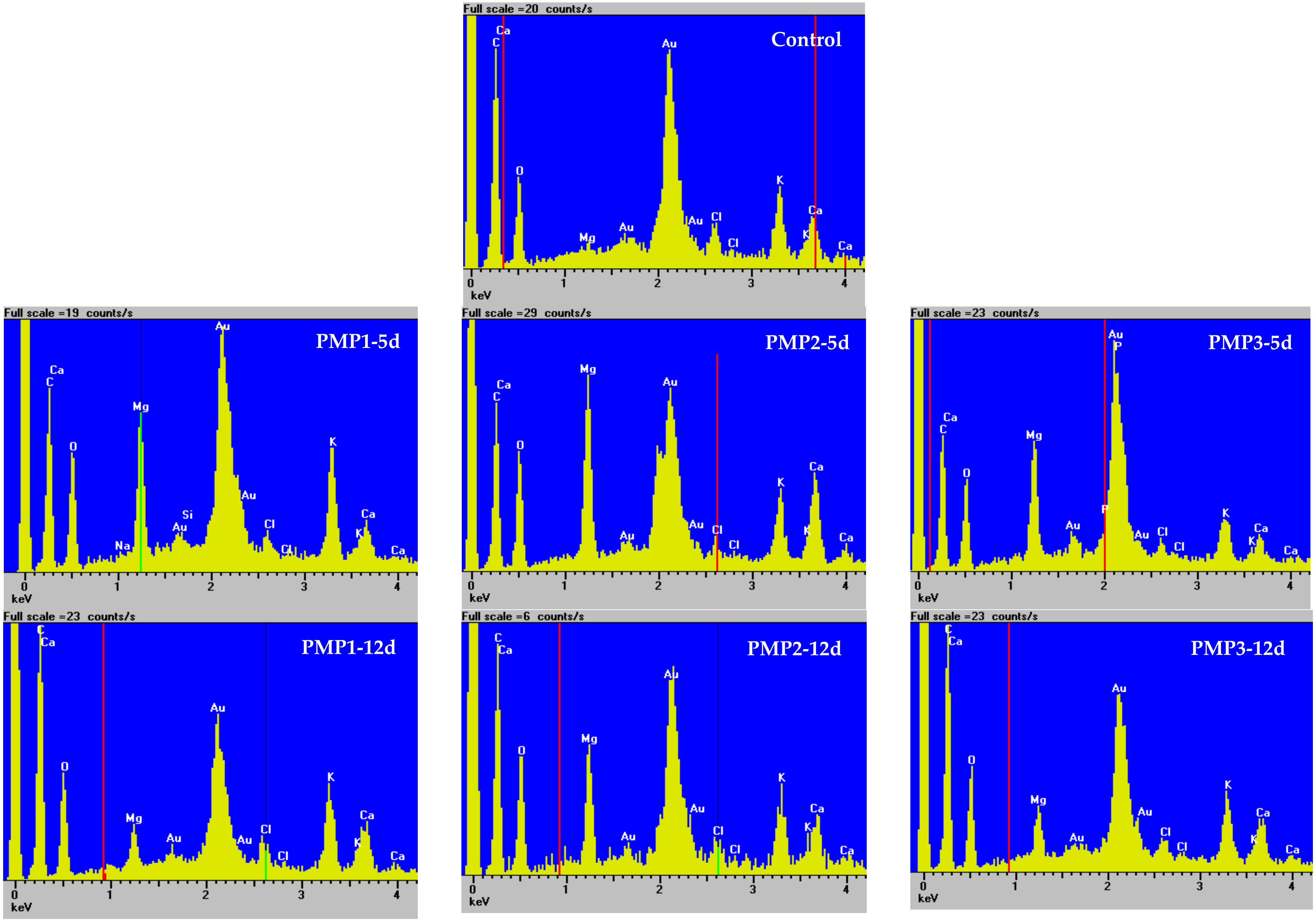

Figure S1 Images from Energy Dispersive X-Ray Spectroscopy carried out during SEM analysis of sprayed tomato leaves shown in Figure 6 of the manuscript.

1. Song, Y.; Chen, D.; Lu, K.; Sun, Z.; Zeng, R. Enhanced tomato disease resistance primed by arbuscular mycorrhizal fungus. *Frontiers in Plant Science* **2015**, *6*, doi:10.3389/fpls.2015.00786.
2. Tan, X.L.; Chen, J.L.; Benelli, G.; Desneux, N.; Yang, X.Q.; Liu, T.X.; Ge, F. Pre-infestation of Tomato Plants by Aphids Modulates Transmission-Acquisition Relationship among Whiteflies, Tomato Yellow Leaf Curl Virus (TYLCV) and Plants. *Frontiers in Plant Science* **2017**, *8*, doi:10.3389/fpls.2017.01597.
3. Balaji, V.; Mayrose, M.; Sherf, O.; Jacob-Hirsch, J.; Eichenlaub, R.; Iraki, N.; Manulis-Sasson, S.; Rechavi, G.; Barash, I.; Sessa, G. Tomato Transcriptional Changes in Response to Clavibacter michiganensis subsp. michiganensis Reveal a Role for Ethylene in Disease Development. *Plant Physiology* **2008**, *146*, 1797-1809, doi:10.1104/pp.107.115188.
4. Aimé, S.; Alabouvette, C.; Steinberg, C.; Olivain, C. The Endophytic Strain Fusarium oxysporum Fo47: A Good Candidate for Priming the Defense Responses in Tomato Roots. *Molecular Plant-Microbe Interactions®* **2013**, *26*, 918-926, doi:10.1094/mpmi-12-12-0290-r.
5. Tsaballa, A.; Nikolaidis, A.; Trikka, F.; Ignea, C.; Kampranis, S.C.; Makris, A.M.; Argiriou, A. Use of the de novo transcriptome analysis of silver-leaf nightshade (Solanum elaeagnifolium) to identify gene expression changes associated with wounding and terpene biosynthesis. *BMC Genomics* **2015**, *16*, 504, doi:10.1186/s12864-015-1738-3.
